# Modifications in Wheelchair Propulsion Technique with Speed

**DOI:** 10.3389/fbioe.2015.00171

**Published:** 2015-10-26

**Authors:** Ian M. Russell, Shashank Raina, Philip S. Requejo, Rand R. Wilcox, Sara Mulroy, Jill L. McNitt-Gray

**Affiliations:** ^1^Department of Biomedical Engineering, University of Southern California, Los Angeles, CA, USA; ^2^Pathokinesiology Laboratory, Rancho Los Amigos National Rehabilitation Center, Downey, CA, USA; ^3^Department of Psychology, University of Southern California, Los Angeles, CA, USA; ^4^Department of Biological Sciences, University of Southern California, Los Angeles, CA, USA

**Keywords:** biomechanics, spinal cord injury, shoulder pain, wheelchair, rehabilitation, propulsion, joint kinetics, upper extremity

## Abstract

**Objective:**

Repetitive loading of the upper limb joints during manual wheelchair (WC) propulsion (WCP) has been identified as a factor that contributes to shoulder pain, leading to loss of independence and decreased quality of life. The purpose of this study was to determine how individual manual WC users with paraplegia modify propulsion mechanics to accommodate expected increases in reaction forces (RFs) generated at the pushrim with self-selected increases in WCP speed.

**Methods:**

Upper extremity kinematics and pushrim RFs were measured for 40 experienced manual WC users with paraplegia while propelling on a stationary ergometer at self-selected free and fast propulsion speeds. Upper extremity kinematics and kinetics were compared within subject between propulsion speeds. Between group and within-subject differences were determined (α = 0.05).

**Results:**

Increased propulsion speed was accompanied by increases in RF magnitude (22 of 40, >10 N) and shoulder net joint moment (NJM, 15 of 40, >10 Nm) and decreases in pushrim contact duration. Within-subject comparison indicated that 27% of participants modified their WCP mechanics with increases in speed by regulating RF orientation relative to the upper extremity segments.

**Conclusions:**

Reorientation of the RF relative to the upper extremity segments can be used as an effective strategy for mitigating rotational demands (NJM) imposed on the shoulder at increased propulsion speeds. Identification of propulsion strategies that individuals can use to effectively accommodate for increases in RFs is an important step toward preserving musculoskeletal health of the shoulder and improving health-related quality of life.

## Introduction

Preserving shoulder function in individuals with spinal cord injury (SCI) continues to be a significant problem (Gutierrez et al., 2007; Alm et al., 2008). Effective interaction between an individual and their manual wheelchair (WC) is essential to preserving quality of life, specifically shoulder function and overall health (Curtis et al., 1999; Gutierrez et al., 2007). Although the clinical problem of shoulder pain in individuals with SCI was identified more than three decades ago, the prevalence remains high (Silfverskiold and Waters, 1991; Pentland and Twomey, 1994; Jain et al., 2010). Researchers and clinicians have attributed shoulder pain in the SCI population to the repetitive mechanical loading of the upper limb as a consequence of lower extremity paralysis (Bayley et al., 1987; Dalyan et al., 1999). In individuals with paraplegia, shoulder pain can occur within the first year and the incidence increases with time post-injury (35% at 5 years, 70% at 20 years, Sie et al., 1992). Due to the detrimental impact on functional mobility and the difficulty in treatment of shoulder pain, effective preventative strategies must be determined for *each* WC user. The activities that provoke the highest pain responses for full-time manual WC users tend to be those that are repetitive and generate high shoulder forces, such as manual wheelchair propulsion (WCP) (Curtis et al., 1999).

Manual WCP is a cyclic task that requires repetitive generation of propulsive forces on the pushrim of the WC. Generation of these reaction forces (RFs) applied at the pushrim involves coordinated activation of muscles responsible for simultaneously maintaining shoulder joint stability and controlling shoulder rotation. Structural stability of the shoulder joint is provided by a shallow humeral head socket (glenoid cavity) and a fibrous labrum (Inman et al., 1944). During WCP, the elbow is positioned below the shoulder. In this segment configuration, the joint capsule tends to be loose and the reinforcing ligaments are slack in absence of a RF, thereby creating the need for shoulder muscles to maintain joint stability (Mulroy et al., 1996). Simultaneously, activation of the upper extremity muscles must be coordinated to produce the shoulder and elbow net joint moments (NJMs) needed to generate propulsive RFs on the pushrim (Robertson et al., 1996; Kulig et al., 1998; Koontz et al., 2002). Imposing both joint stability and moment generation requirements on muscles in the shoulder region during WCP increases the susceptibility to neuromuscular fatigue (Kulig et al., 1998; Koontz et al., 2002). A weakened muscle within the shoulder girdle complex can result in an inadequate dynamic stability of the shoulder particularly during intervals when large RFs are required during WCP (McCully et al., 2007). Loss of dynamic stability causes stress on the shoulder structures and other joints of the upper limb and can lead to the development of shoulder pain (Curtis et al., 1999; Gironda et al., 2004; Samuelsson et al., 2004; Alm et al., 2008).

As part of daily living, manual WC users need to regulate WCP speed. On average, increases in WCP speed has been reported to significantly increase RF magnitudes, decrease hand contact duration, affect wrist angular position on pushrim (Kulig et al., 1998; Koontz et al., 2002; Veeger et al., 2002), and influence the mechanical demand imposed on muscles controlling shoulder stabilization and rotation during WCP (Kulig et al., 1998; Koontz et al., 2002). Increases in WCP speed can also lead to disproportionate increases in shoulder NJMs during hand contact (Veeger et al., 2002). Understanding how an individual can effectively interact with the pushrim to achieve required increases in WCP speed provides insights into how modifications in multijoint control of the upper limb can accommodate for increased mechanical demand imposed on the shoulder. Model simulation results indicate that modifications in RF orientation relative to the upper extremity segments can effectively redistribute load away from the shoulder while maintaining WCP speed (Munaretto et al., 2012, 2013). To date, the techniques used by individuals with SCI to accomplish the changes in propulsion speeds have been difficult to discern from group mean data of peak NJMs reported during WCP (Kulig et al., 1998, 2001; Koontz et al., 2002; Mercer et al., 2006).

In this study, we used a within-subject experimental design to determine how individual manual WC users with paraplegia modify WCP mechanics to accommodate expected increases in RF generated at the pushrim with self-selected increases in propulsion speed. As found previously, we expect that RF magnitude, shoulder net joint force (NJF), and shoulder NJM during WCP would increase whereas contact duration would decrease with increases in speed (Kulig et al., 1998; Koontz et al., 2002; Veeger et al., 2002). Consistent with that found in other impulse generating tasks (McNitt-Gray et al., 2001; Mathiyakom et al., 2005) and experimental-based model simulations of WCP (Munaretto et al., 2012), we hypothesized that the orientation of RF relative to the forearm and upper arm would affect the mechanical demand imposed on the upper extremity with increases in WCP speed. We anticipated that individuals with paraplegia would use different WCP techniques to accommodate the need to increase WCP speed. Modifications in WCP technique between free and self-selected fast WCP speeds were characterized by identifying within-subject differences in upper extremity joint kinetics at peak push during hand contact with the pushrim. Identification of effective load distribution strategies that an individual can use during manual WCP at different speeds provides evidence to support clinical decisions as to how and when to explore modifications in WCP technique as a means of preserving shoulder function in individuals with SCI.

## Materials and Methods

### Participants

Forty participants (32 male and eight female) with complete SCI who were experienced manual WC users with paraplegia (T2-L3) from the outpatient clinics of the Rancho Los Amigos National Rehabilitation Center volunteered to participate. Each participant was provided informed consent in accordance with the Institutional Review Board. Individuals were excluded from participation if they reported a history of shoulder pain that altered performance of daily activities or required medical treatment. Average (SD) weight of participants was 74.5 (18) kg, average height was 1.73 (0.1) m and average age was 35 years (range: 18–62 years). The mean time since occurrence of the injury was 8.25 years (range: 2–20 years).

### Instrumentation

For this study, the majority of the participants propelled their own WC using an ergometer (27 of 40). In cases when the individual’s WC did not fit the ergometer set-up (13 of 40), the individual used a rigid frame, lightweight Quickie GPV WC with either a 16″ or 18″ seat, depending on the size of the participant. Horizontal and vertical axle positions were matched to that of the individual’s WC. The height of the footrest, seat back, and inertial parameter of the test WC were also adjusted to match the participant’s own WC. Each participant used their own seat cushion. The WC was positioned on a stationary ergometer, consisting of a support frame and split rollers, allowing separate rotation of each wheel. The rollers were coupled by means of a differential to an alternator and a modified Velodyne^®^ bicycle ergometer that computer-controlled the resistance. To quantify the friction force between the tire and ergometer rollers, a coast down test (from 182 to 35 m/min) with the participant sitting in the test WC on top of the ergometer was used. Removable flywheels proportional to the weight of both the person and the WC were used to simulate the translational inertia of “over ground” propulsion. Further details about the ergometer instrumentation and calibration steps are described in previous papers (Mulroy et al., 2004, 2005; Requejo et al., 2008; Lighthall-Haubert et al., 2009). RF applied by the hand to the pushrim was measured using three strain gage force transducers at 200 Hz (SmartWheel, Three Rivers Holdings, Mesa, AZ, USA).

### Data Collection

Three-dimensional trunk, right-side upper extremity and wheel kinematics were collected with active infra-red markers using a CODA motion analysis system (6-camera, CODA Motion Analysis system, 100 Hz) for 10 s of WCP at two speed conditions. Markers were placed on the trunk at the manubrium, the xiphoid process, the spinous process of T3 and T10 vertebrae, greater tubercle of the humerus, lateral epicondyle, medial epicondyle, deltoid tuberosity, middle of the forearm, radial styloid, ulnar styloid, head of the third metacarpal, and head of the fifth metacarpal. Three reflective markers were also placed on the right wheel.

### Experimental Protocol

Prior to data collection, participants were given adequate time to become accustomed to the WC and experimental conditions. Each participant performed WCP at their self-selected free speed, as they do normally when traversing a tiled floor, and at a self-selected fast speed, as if they are in a hurry to not miss an important appointment. Preceding the start of data collection, participants propelled for 30 s to avoid the propulsion initiation period. Force and kinematic data were then collected for 10 s (6–10 push cycles) at each speed condition with no additional load applied to the ergometer rollers (i.e., level ground over a tiled surface).

### Data Processing and Analysis

The kinematic and force data of consecutive propulsion cycles during the data collection interval (10 s) were analyzed using Visual3D^d^ and Matlab^f^. The number of propulsion cycles analyzed for each subject was the maximum number of propulsion cycles captured in the 10-s window common to all subjects for that condition (5 for free and 6 for fast). Kinematic data were filtered in Visual3D using a sixth order low-pass filter with a cutoff frequency of 8 Hz (Cooper et al., 2002). Four segments were constructed based on the ISB standard definitions (Wu et al., 2005). The thorax segment was defined using markers placed at the xiphoid, manubrium, T3, and T10 vertebrae. The upper arm segment was constructed with the marker at the humeral head, a non-collinear marker on the upper arm, and the lateral humeral epicondyle marker. The forearm segment was created using the lateral humeral epicondyle marker, a non-collinear marker on the forearm, and the marker on the ulnar styloid process. The hand segment was created using the markers of the radial styloid, ulnar styloid, the head of the third metacarpal. Segment inertia parameters were based on body segment parameters (de Leva, 1996).

**Figure 1:**
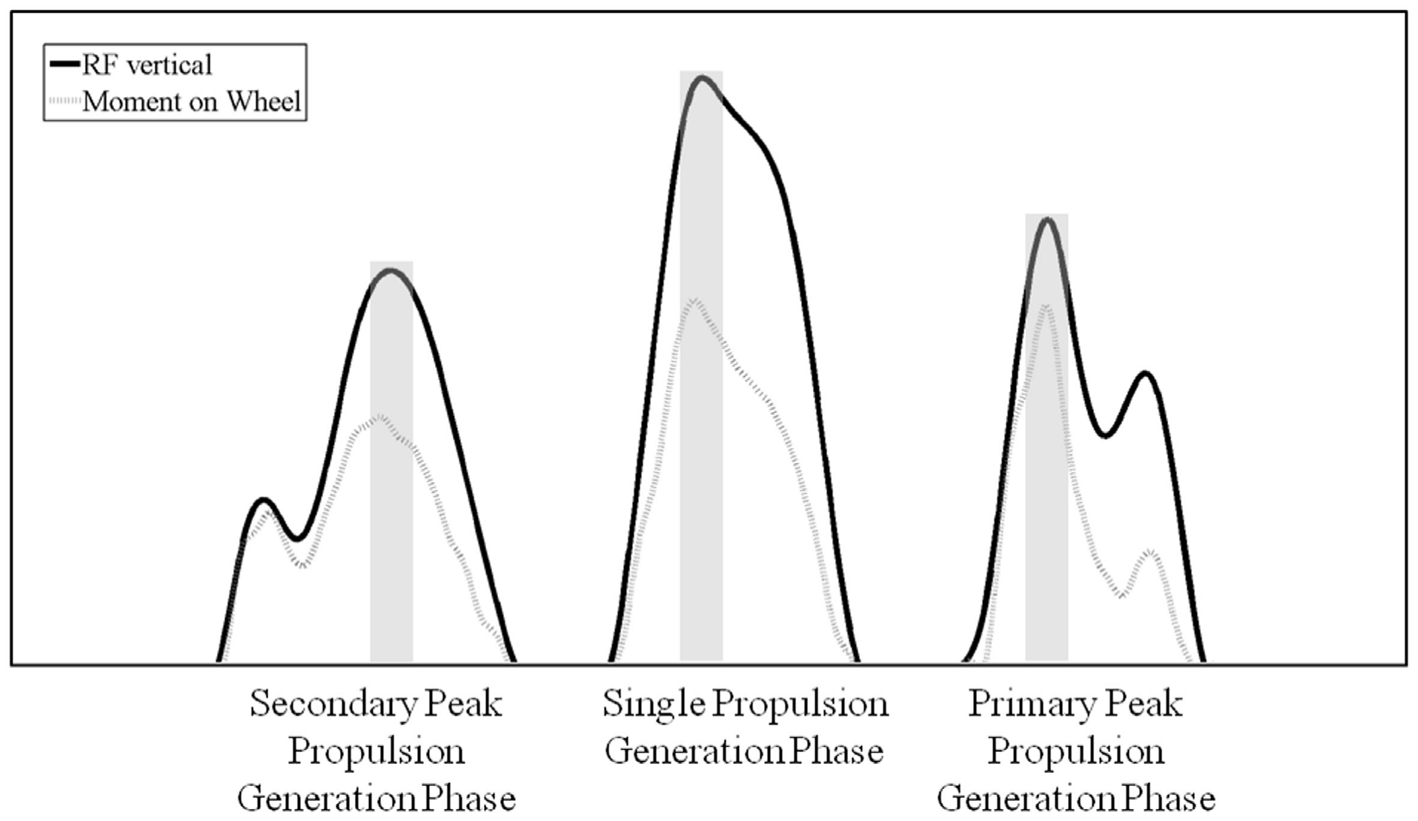
**Vertical reaction force and moment on the wheel for three example propulsion cycles illustrating the three different propulsion strategies seen in the data**. The shaded regions show the duration around peak averaged to define peak push.

Cycle duration, defined as the elapsed time between successive hand-pushrim contacts, was determined using measured pushrim RF data. Contact phase of the cycle was defined from the point in time when the vertical component of the RF exceeded 3 N to the time of rim release, when the RF reduced to below 3 N. To characterize differences in initiation of hand contact with the pushrim and propulsion generation strategies between individuals, the number of peaks in RF observed during the contact phase were noted (Figure 1). The contact phase was further divided into sub-phases: the impact (IP) phase when present and a propulsion-generating phase(s) (PGP). The IP was defined as the interval immediately after pushrim contact (from initial hand contact to time of next local minimum) and was typically not associated with substantial torque acting to rotate the wheel. Time of peak push was identified as the time of the maximum peak in the vertical RF measured during PGP.

Kinematic and RF at the pushrim were synchronized at time of initial contact with the pushrim and used to calculate 3D NJM and NJF at the elbow and shoulder (100 Hz) using inverse dynamics in Visual3D. The magnitudes of the RF, NJF, and NJM at the elbow and shoulder are reported for peak push as the average of the six points around the peak in vertical RF during the PGP. The relative contribution of the elbow and shoulder to the mechanical demand imposed on the upper extremity was determined for peak push by the NJM at each joint divided by the sum of the NJMs at both joints (shoulder and elbow). The orientation of the RF relative to the forearm and upper arm was expressed by the angle of the resultant RF projected into the arm plane (created by the wrist, elbow, and shoulder).

### Statistics

The probabilities for each variable being less during the free condition than the fast condition when comparing across propulsion conditions was calculated using a Sign Test. Assuming local independence for trials and that the free and fast conditions were independent for each subject, these comparisons were repeated for each variable for within-subject statistical significance as well (R, open-source). A *p*-value was then calculated for each subject using Cliff’s analog of the Wilcoxon–Mann–Whitney test (Cliff, 1996). A modified, step-down Fisher-type method was then applied to control the familywise error rate of α = 0.05 over multiple comparisons (Wilcox and Clark, 2015). This within-subject analysis was used to determine which subjects had statistically significant changes when comparing their self-selected free propulsion cycles to their self-selected fast propulsion cycles.

## Results

**Figure 2:**
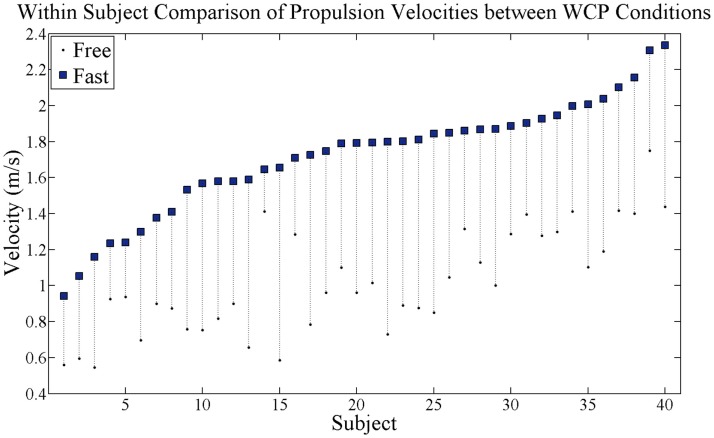
**Within-subject comparison of self-selected wheelchair propulsion velocity**. Black dots are velocity at free speed condition and blue squares are velocity at fast speed condition. Dotted vertical lines connect each subject’s free and fast velocities and show velocity increase. All subjects successfully increased propulsion velocity.

Consistent with the experimental design, all of the 40 participants significantly increased their WCP speed between free and fast conditions across all participants (*p* = 0.0001, Figure 2). Mean velocity across all participants during free condition was 1.02 m/s (0.3) and mean velocity across all participants during fast condition was 1.72 m/s (0.3). The velocity increase between free and fast conditions was on average 0.70 (0.2) m/s across participants.

**Figure 3:**
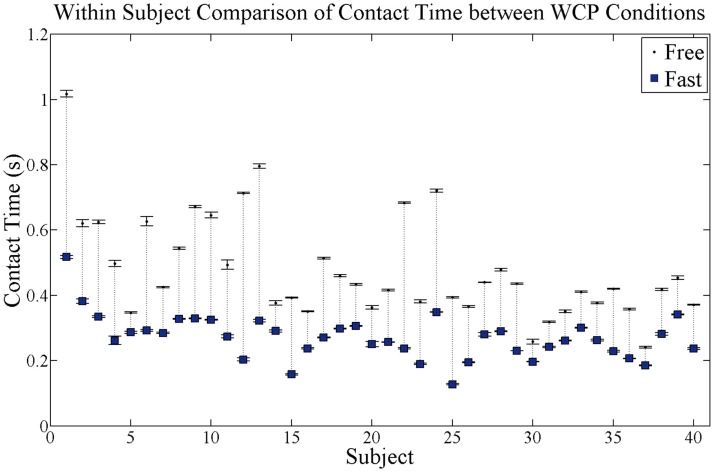
**Within-subject comparison of average contact time for each subject for both self-selected free and fast speed conditions**. Black dots are contact time at free speed condition and blue squares are contact time at fast speed condition. SE bars are shown for both conditions. Dotted vertical lines connect each subject’s free and fast contact times and show magnitude of the change in contact time. Within-subject comparison found 32 of the 40 participants significantly reduced contact duration.

As expected, hand-rim contact duration significantly decreased with increases in WCP speed across all participants (*p* = 0.0001, Figure 3). Within-subject comparisons indicated that 39 of the 40 participants reduced contact duration with increases in WCP speed. Of those 39 participants, 18 reduced contact duration by 0.20 s or more.

**Figure 4:**
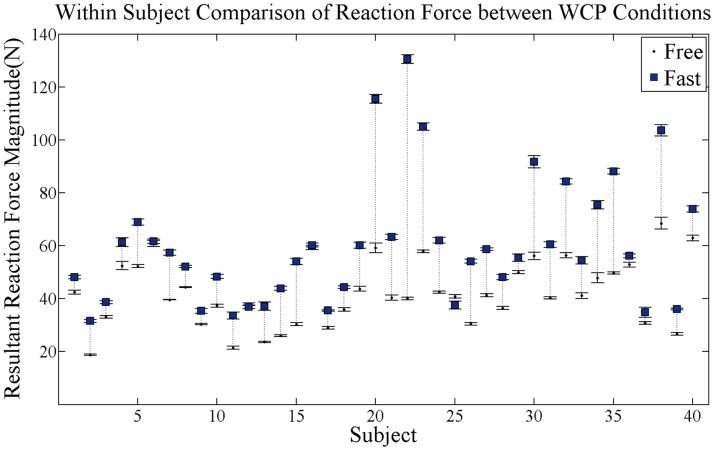
**Within-subject comparison of average resultant RF magnitude at peak push for each subject for both self-selected free and fast speed conditions**. Black dots are average RF magnitude at free speed condition and blue squares are average RF magnitude at fast speed condition. SE bars are shown for both conditions. Dotted vertical lines connect each subject’s free and fast RF magnitudes and show magnitude change in RF. Within-subject comparison found 26 of the 40 participants increased resultant RF at peak push.

The resultant RF magnitude at peak push significantly increased for the fast as compared to the free WCP condition across all participants (*p* = 0.0001) (Figure 4). Within-subject comparisons indicated that 26 of the 40 participants increased resultant RF at peak push between the free and fast conditions. Of those 26 participants, 22 increased the resultant RF by 10 N or more.

**Figure 5:**
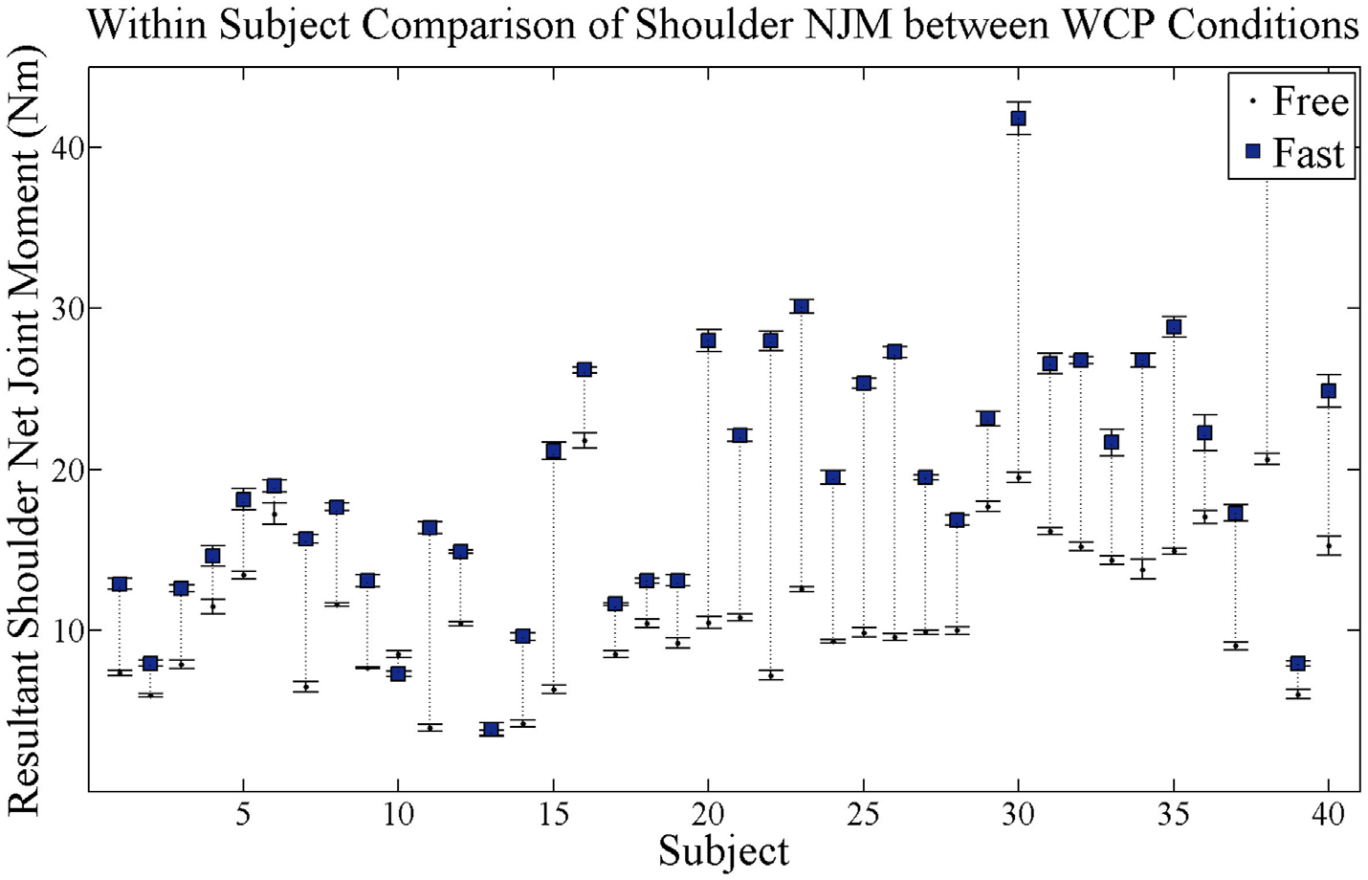
**Within-subject comparison of average resultant NJM magnitude on the shoulder at peak push for each subject for both self-selected free and fast speed conditions**. Black dots are average shoulder NJM magnitude at free speed condition and blue squares are average shoulder NJM magnitude at fast speed condition. SE bars are shown for both conditions. Dotted vertical lines connect each subject’s free and fast NJM magnitudes and show magnitude change in NJM. Within-subject comparison revealed that 30 of 40 participants showed a significant increase in resultant NJM on the shoulder with increases in WCP speed.

The resultant shoulder NJM at peak push significantly increased in the fast as compared to free WCP conditions across all participants (p = 0.0001) (Figure 5). Within-subject comparison revealed that 30 of 40 participants increased resultant shoulder NJM with increases in WCP speed, with 15 participants increasing shoulder NJM by 10 Nm or more.

**Figure 6:**
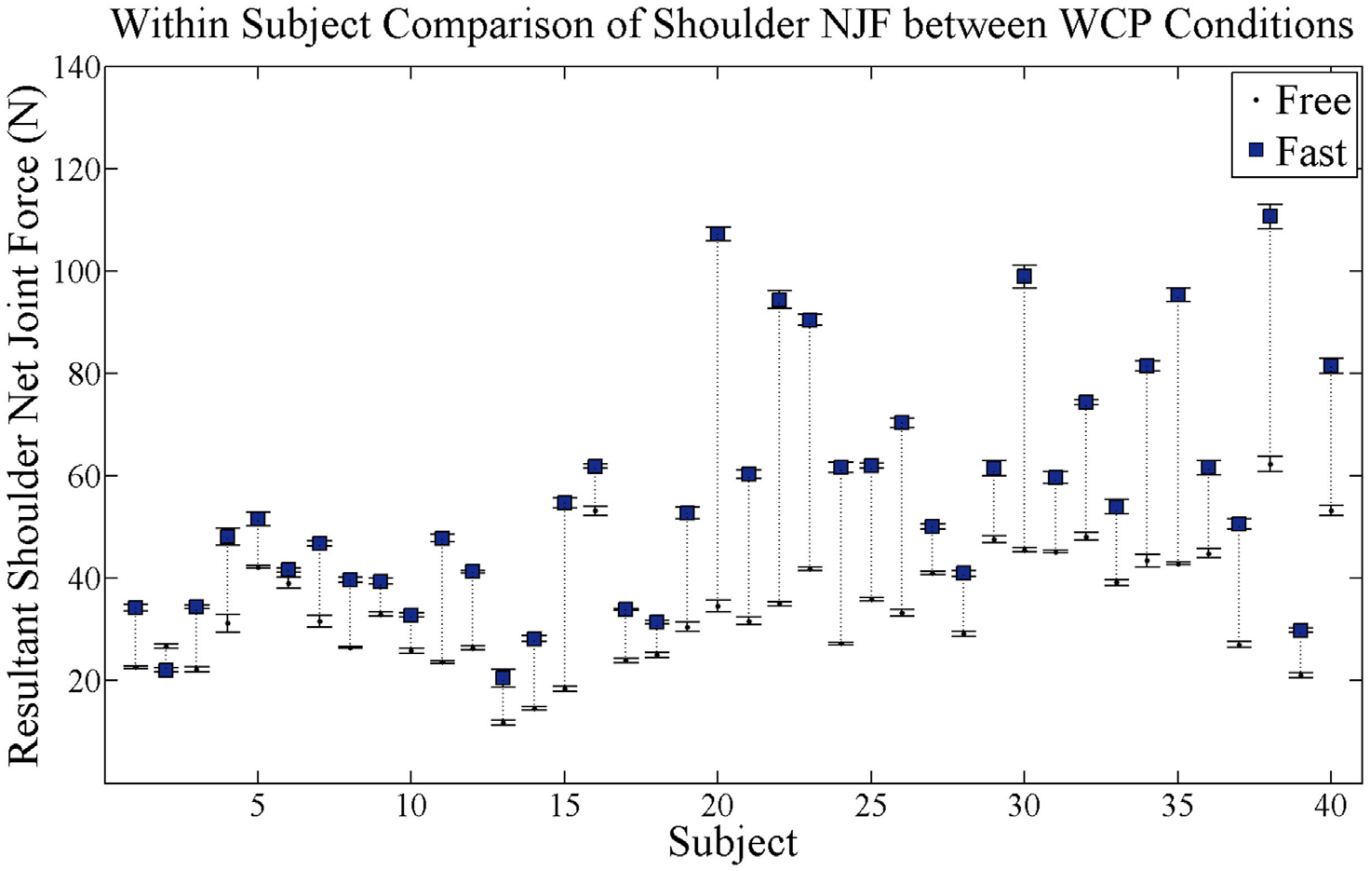
**Within-subject comparison of average resultant shoulder NJF magnitude at peak push for each subject for both self-selected free and fast speed conditions**. Black dots are average shoulder NJF magnitude at free speed condition and blue squares are average shoulder NJF magnitude at fast speed condition. SE bars are shown for both conditions. Dotted vertical lines connect each subject’s free and fast NJF magnitudes and show magnitude change in NJF.

The resultant shoulder NJF at the time of peak push significantly increased in the fast as compared to free WCP conditions across all participants (*p* = 0.0001) (Figure 6). On average, resultant shoulder NJF increased by 23 N when propelling under the fast as compared to the free WCP condition.

**Figure 7:**
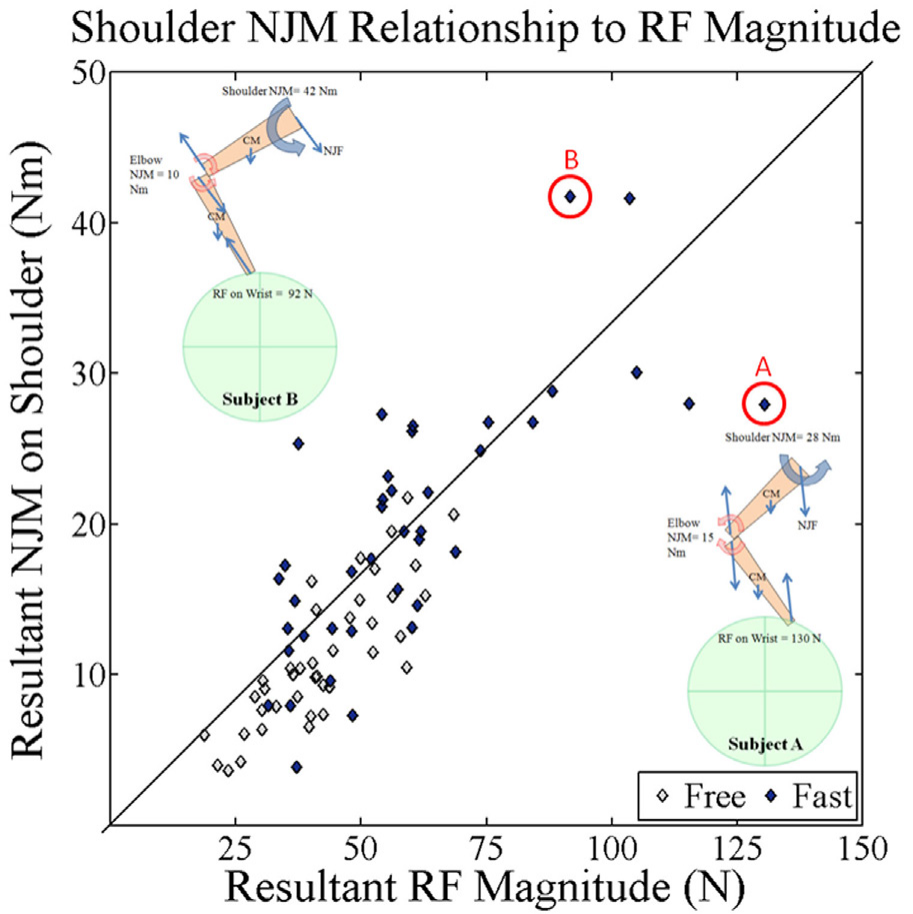
**Average resultant NJM magnitude on the shoulder at peak push for each subject for both self-selected free and fast speed conditions plotted against average resultant RF magnitude at peak push for each subject**. Diagonal line represents a 1:1 relationship, meaning that a twofold increase in RF would lead to a twofold increase in NJM on the shoulder. At the higher RF magnitudes, a few subjects deviate further from this relationship. Subjects A and B illustrate how RF orientation relative to upper extremity can affect shoulder NJMs relationship to RF magnitude.

As hypothesized, orientation of RF relative to the forearm and upper arm affected the mechanical demand imposed on the upper extremity with increases in WCP speed. Increases in RF magnitude did not necessarily result in proportionate increases in shoulder NJM within subject (Figure 7). For example, in the fast WCP condition, subjects A and B both generated relatively large RFs (130 and 92 N, respectively) but different techniques led to different magnitudes in shoulder NJMs (Figure 7). Subject A oriented the RF anterior to forearm resulting in an elbow extensor NJM and a shoulder flexor NJM of 28 Nm. In contrast, Subject B RF was more aligned with the forearm resulting in an elbow flexor NJM and a shoulder flexor NJM of 41 Nm.

Increase is WCP speed, from free to self-selected fast, was accomplished using different techniques within subject. In some cases, increases in WCP speed were associated with significant increase in RF magnitude without modifications in upper extremity kinematics (12 of 40). In other cases, individuals significantly modified RF orientation, forearm orientation, or both, resulting in modifications in mechanical demand imposed on the shoulder. More vertical orientations of the forearm at peak push was associated with hand positions more posterior on the pushrim, whereas more horizontal orientation of the forearm at peak push was associated with hand positions that were more anterior on the pushrim. No significant within-subject differences in elbow angle at peak push were noted between WCP speeds, suggesting muscle lengths were maintained across WCP conditions.

**Figure 8:**
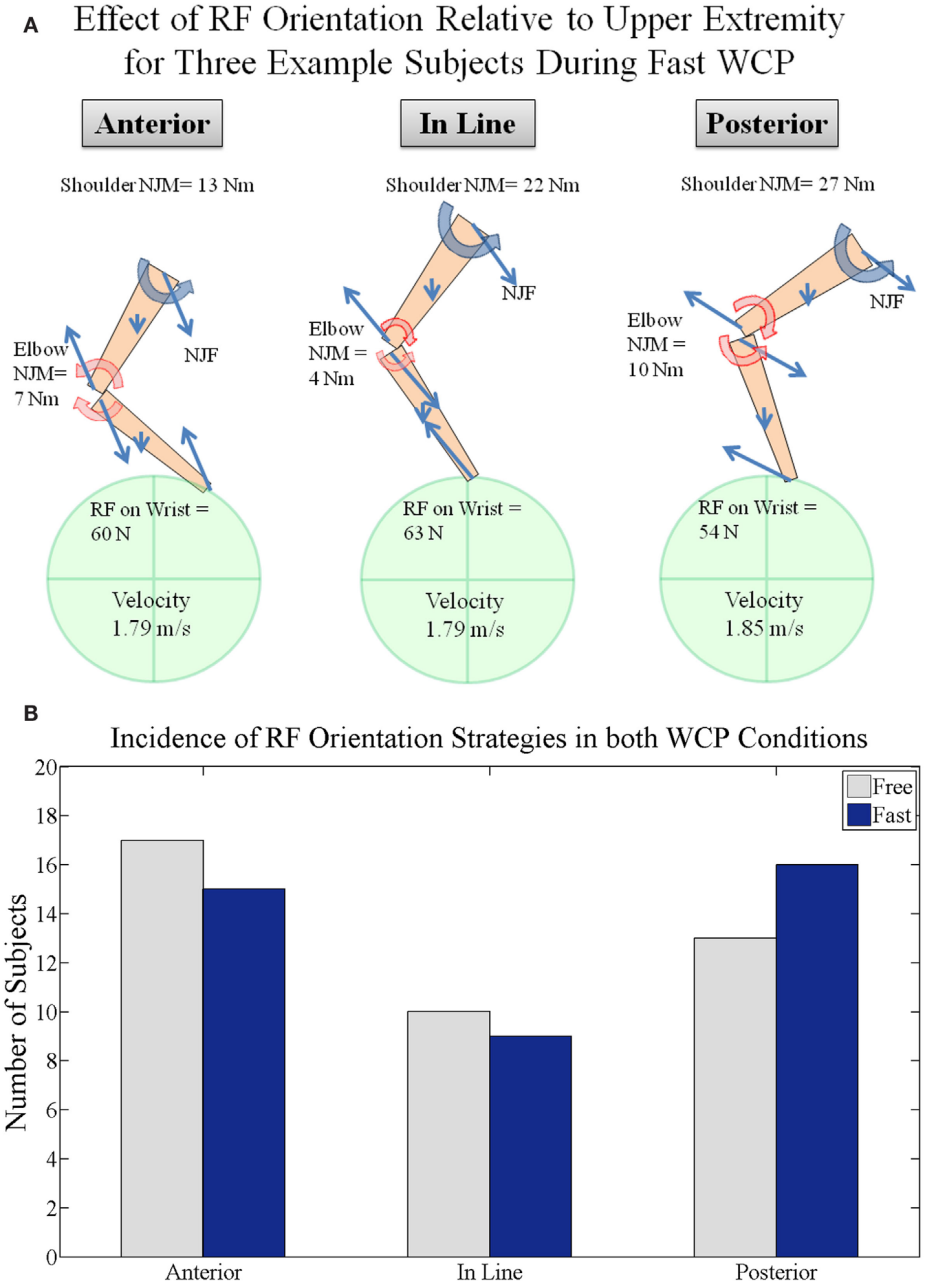
**(A)** Effect of RF orientation relative to the upper extremity segments for three example subjects with comparable propulsion velocities and RF magnitudes. Free body diagrams are drawn for fast speed condition at the time of peak push. Note elbow NJMs are in opposite directions for the anterior and posterior examples and how that affects shoulder NJM. **(B)** Population grouping of RF component orientation in the armplane (plane that connects shoulder, elbow, and wrist) relative to the upper extremity at the time of peak push. Orientation is grouped into posterior (more than 5° behind the forearm), anterior (more than 5° in front of the forearm), and in line (within 5° posterior or anterior).

In some cases, individuals were able to mitigate increases in the rotational demand imposed on the shoulder with increases in WCP speed, whereas others were not. For example, the three exemplar participants achieved comparable fast WCP velocities with comparable RF magnitudes at peak push (Figure 8A). However, the magnitude of the shoulder NJM depended on the proximal distal moments created by the NJFs about the center of mass (CM) of the forearm and upper arm segments as well as the adjacent joint NJM at the elbow. When the RF is oriented *anterior* to the forearm CM, an elbow extensor NJM is needed to achieve the observed motion. The elbow extensor NJM applied to the upper arm contributes to the reduction in magnitude of the shoulder NJM. In contrast, when the RF is oriented *posterior* to the forearm CM, an elbow flexor NJM is needed to achieve the observed motion. The elbow flexor NJM applied to the upper arm contributes to the increase in magnitude of the shoulder NJM.

In the free WCP condition, the RF orientation relative to the forearm CM at peak push varied across all participants [anterior (17), aligned (10 within 5°), posterior (13), Figure 8B]. Likewise, in the fast propulsion condition, the RF orientation relative to the forearm CM at peak push tended to be evenly distributed across all participants [anterior (15), aligned (9 within 5°), posterior (16)].

Within-subject comparison in RF orientation relative to the forearm CM at peak push indicated that shifts in orientation varied with WCP speed. Within-subject analysis indicated 11 of 40 participants made a significant shift in RF orientation relative to the forearm at peak push when increasing WCP speed. Six of 11 shifted RF in a direction consistent with increasing the shoulder NJM (Figure 8A), while five of 11 participants shifted RF in a direction consistent with decreasing the shoulder NJM. Nine of 11 participants modified the RF orientation relative to the forearm by more than 10°.

On an average, there were no consistent shifts across all participants in distribution of the total arm moment across the upper extremity when increasing WCP speed. Within-subject comparisons indicated that 10 of 40 participants showed a significant increase in the relative contribution of resultant shoulder NJM to the total arm moment. The largest shift in load distribution (reduction in shoulder NJM contribution to total arm moment by 30%) was accomplished by orienting RF more anterior to forearm (13–27°) and more aligned with the upper arm (28°).

No significant shifts in RF alignment with the arm plane at peak push were observed between WCP conditions across all participants. Within-subject analysis revealed that five of the 40 participants showed a statistically significant shift in RF alignment (re-alignment of RF relative to arm plane >5%) with increases in WCP speed. The RF was less aligned with the arm plane for four of five of those participants, thereby contributing to out of plane shoulder NJMs.

## Discussion

During daily activities, manual WC users often encounter situations that result in increases in the mechanical demand imposed on the upper extremity, such as speeding up, going up ramps, or traversing carpets and grass. Understanding the different techniques individual’s use during tasks with increased upper extremity demands is important for identifying manual WCP strategies that can help preserve shoulder function, maintain independence, and improve quality of life. The results of this study indicate that increases in RF magnitudes associated with increases in WCP speed do not necessarily translate into comparable increases in shoulder NJMs. The magnitude of the shoulder NJM depends on the proximal distal moments created by the NJFs about the CM of the forearm and upper arm segments as well as the adjacent joint NJM at the elbow. Within-subject analysis indicated more than 25% of the participants made a significant shift in RF orientation relative to the forearm at peak push when increasing WCP speed. In approximately half of these cases, reorienting the RF relative to the upper extremity segments was used as an effective strategy for mitigating rotational mechanical demand imposed on the shoulder at increased WCP speeds. In the other cases, the shift in RF orientation relative to the forearm at peak push at increased WCP speeds contributed to increases in the shoulder NJM and reductions in the vertical component of the shoulder NJF. By investigating WCP technique modifications in response to increases in WCP speed using a within-subject design, preferential shifts in mechanical loading imposed on the shoulder can be determined. This knowledge of self-selected load mitigation strategies may prove fruitful in guiding clinical decisions that aim to identify strategies for preserving shoulder function in individuals with SCI.

The self-selected free and fast propulsion velocities attained in our sample population are comparable to those found in Kulig et al. (1998). Joint kinetics in this study was also found to be in line with magnitudes previously reported in the literature (Kulig et al., 1998; Koontz et al., 2002; Veeger et al., 2002; Collinger et al., 2008). In this study, an ergometer was used to achieve self-selected steady-state WCP speeds for multiple cycles. To minimize limitations associated with this experimental set-up, a within-subject design was used as a means for each individual to serve as their own control. Consistent with previously reported group mean data (Kulig et al., 1998; Koontz et al., 2002; Veeger et al., 2002; Collinger et al., 2008), the resultant RF as well as the resultant shoulder NJM and NJF at peak push significantly increased in the fast WCP speed condition when compared to free WCP across subjects.

In order to increase WCP speed, the tangential component of the RF being applied to the pushrim must increase in magnitude, particularly if the pushrim contact duration decreases with WCP speed. The participants in this study increased WCP speed using a variety of different techniques. Some participants increased WCP speed by amplifying RF magnitude without modifications in upper extremity kinematics. Whereas other individuals significantly modified RF orientation, forearm orientation, or both, resulting in modifications in mechanical demand imposed on the shoulder. Minimal changes in elbow angle at peak push were observed across speeds, suggesting individuals may have chosen to maintain a preferred muscle length when generating RF at peak push. Results of this study illustrated how choice of orientation of RF relative to the upper extremity affected mechanical demand on the shoulder. Orientation of RF anterior to the forearm CM created an elbow extensor NJM, which contributed to a reduction in shoulder NJM magnitude. Conversely, when RF was oriented posterior to the forearm CM the resulting elbow flexor NJM contributed to an increase in shoulder NJM magnitude. These results suggest that individuals choosing to modify WCP technique by shifting the RF more anterior to the forearm CM may favor reductions in shoulder NJM over increases in the vertical component of the RF, and vice versa. Identification of preferences toward a particular load mitigation strategy may prove fruitful in guiding clinical decisions that aim to identify strategies for preserving shoulder function in individuals with SCI.

The experimental results of this study are consistent with the model simulation results (Munaretto et al., 2012, 2013) that demonstrate at a particular WCP speed, increases in resultant pushrim RF can occur without comparable increase in shoulder NJM. The magnitude of the shoulder NJM is dependent on the proximal distal moments created by the NJFs at the elbow and shoulder and the elbow NJM (Figure 8). The magnitude of the proximal and distal moments is dependent on the magnitude of the NJFs and their orientation relative the upper arm. Redirection of the RF relative to the upper extremity, as shown in both the experimental and model simulation results, can serve as a potential strategy to redistribute load imposed on the upper extremity. Simulation results indicate that WCP speed can be maintained with minimal changes in shoulder NJM even if the corresponding RF doubles in magnitude, provided the RF is reoriented relative to the forearm and upper arm. These results indicate that alignment of the RF anterior to the forearm can mitigate the effect of higher pushrim forces on shoulder NJM magnitude. This strategy may prove to be an effective means of redistributing the mechanical loads imposed on the upper extremity joints during WCP.

Maintaining shoulder health requires more than reducing mechanical demand. Certain scapular and glenohumeral orientations have been associated with reducing subacromial space, which increases the potential risk of shoulder impingement syndrome. Previous research by Morrow et al. (2011) and Raina et al. (2012) found that WCP placed the scapula in some of these potentially dangerous orientations that could contribute to the development shoulder impingement. More specifically, Raina’s study showed that with increases in propulsion force, WC user’s scapula tended to move into anterior tilt, downward rotation, and protraction. All of these positions are associated with reduced subacromial space. If this scapular movement occurs in conjunction with upward motion of the humerus in the glenoid cavity, there is potential for impingement of the supraspinatus. The superiorly directed forces transmitted along the axis of the humerus could have a negative long-term consequence if not adequately controlled by muscles crossing the shoulder complex (Mulroy et al., 2005). However, further research must be done with more accurate methods of subacromial space estimation to see if the scapular movement found in WCP is clinically relevant (Raina et al., 2012). Any recommendation in technique modification must consider the ability of the individual to control RF and segment motion during task performance to avoid detrimental loading (McNitt-Gray, 2000).

By examining how individual WC users organized their upper limb coordination to accommodate increases in mechanical demands, effective multijoint control strategies for increasing WCP speed without substantial increases in the shoulder NJM was identified. Future studies will examine how this WCP technique may benefit those with different upper extremity control capabilities and will explore the relative contribution of these factors in regulating shoulder loads during WCP. For individuals unable to modify WCP technique by reorienting pushrim RF relative to the upper extremity segments, customized modifications in WC configuration, including optimal seat height (Kotajarvi et al., 2004), axle horizontal positions (Mulroy et al., 2005), and seat angle (Desroches et al., 2006), are alternative ways of redistributing the mechanical demands within the upper extremity joints.

## Suppliers

- Digital Equipment Corporation, Cambridge, MA, USA.
- Quickie GPV; Sunrise Medical, Quickie Designs Inc., 2842 Business Park Avenue, Fresno, CA 93727, USA.
- Velodyne bicycle ergometer; Schwinn Bicycle Company, 217 N. Jefferson Street, Chicago, IL 60661, USA.
- VICON (Oxford Metrics Ltd, Oxford, England).
- C-Motion, Inc., 15821-A Crabbs Branch Way, Rockville, MD 20855, USA.
- The MathWorks, Inc., 3 Apple Hill Drive, Natick, MA 01760-2098, USA.
- R Programming Language. Link www.r-project.org.

## Conflict of Interest Statement

The authors declare that the research was conducted in the absence of any commercial or financial relationships that could be construed as a potential conflict of interest.
